# Biobased Photopolymer Resin for 3D Printing Containing Dynamic Imine Bonds for Fast Reprocessability

**DOI:** 10.1021/acsami.3c01669

**Published:** 2023-05-23

**Authors:** Jules Stouten, Geraldine H. M. Schnelting, Jerzy Hul, Nick Sijstermans, Kylian Janssen, Tinashe Darikwa, Chongnan Ye, Katja Loos, Vincent S. D. Voet, Katrien V. Bernaerts

**Affiliations:** † Sustainable Polymer Synthesis Group, Aachen-Maastricht Institute for Biobased Materials (AMIBM), Faculty of Science and Engineering, Maastricht University, Brightlands Chemelot Campus, Urmonderbaan 22, 6167 RD Geleen, The Netherlands; ‡ Professorship Circular Plastics, NHL Stenden University of Applied Sciences, van Schaikweg 94, 7811 KL Emmen, The Netherlands; § Liqcreate, Texasdreef 7, 3665 CL Utrecht, The Netherlands; ∥ Macromolecular Chemistry and New Polymeric Materials, Zernike Institute for Advanced Materials, University of Groningen, Nijenborgh 4, 9747 AG Groningen, The Netherlands

**Keywords:** 3D printing, recycling, sustainability, vitrimers, UV curing, polymers

## Abstract

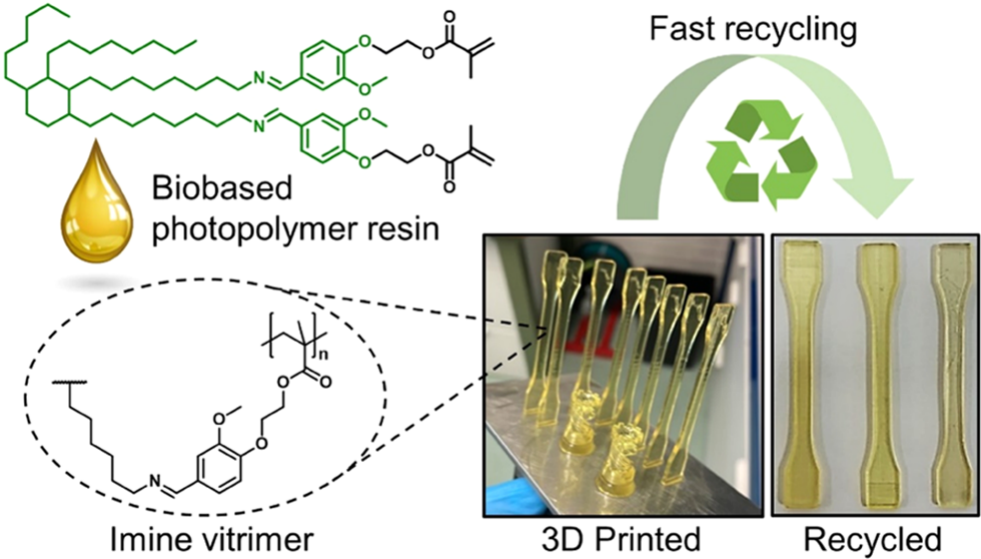

Acrylic photopolymer resins are widely used in stereolithographic 3D printing. However, the growing demand for such thermosetting resins is weighing on global issues such as waste management and fossil fuel consumption. Therefore, there is an increasing demand for reactive components that are biobased and enable recyclability of the resulting thermoset products. In this work, the synthesis of a photo-cross-linkable molecule containing dynamic imine bonds based on biobased vanillin and dimer fatty diamine is described. Using the biobased building blocks, formulations containing reactive diluent and a photoinitiator were prepared. The mixtures could be rapidly cross-linked under UV light, yielding vitrimers. Using digital light processing, 3D-printed parts were prepared, which were rigid, thermally stable, and reprocessed within 5 min at elevated temperature and pressure. The addition of a building block containing a higher concentration of imine bonds accelerated the stress relaxation and improved the mechanical rigidity of the vitrimers. This work will contribute to the development of biobased and recyclable 3D-printed resins to facilitate the transition to a circular economy.

## Introduction

Vat photopolymerization (3D printing) of photopolymer resins is a straightforward method to produce custom-made parts consisting of a thermosetting plastic.^1^ Digital light processing (DLP) is one of the major techniques for the production of 3D-printed objects with excellent dimensional control and resolution.^2^ Liquid acrylic resins employed in DLP are typically composed of molecules containing (meth)acrylate functionality that function as building blocks or reactive diluents, together with a photoinitiator. The resulting products display good mechanical performance, thermal stability, and solvent resistance due to the presence of a cross-linked network. Currently, most commercial resin systems are composed of acrylates derived from fossil resources.^3^ Therefore, it is desirable to increase the availability of cost-competitive biobased alternatives to meet the growing demand for DLP materials and to contribute to the transition to the circular economy.^4−6^

Another problem associated with the rise of DLP is the growing amount of plastic waste consisting of thermoset polymers. A key part of the circular economy is the recycling of plastic materials to avoid disposal in landfills or incineration of plastic waste. One of the major downsides of thermosets is the irreversibility of their networks, rendering them nonrecyclable. Recently, a new class of thermosetting polymers called vitrimers, which make up a subclass of covalent adaptable networks (CANs), has been the subject of thorough investigation.^7,8^ Vitrimers contain cross-links through chemical bonds that are reversible by thermal activation. Therefore, vitrimers can be reprocessed at elevated temperatures while maintaining the good properties of thermosets at the working temperature. Since dynamic exchange in vitrimers proceeds via an associative mechanism, the mechanical and structural stability is improved compared to CANs that show bond exchange via a dissociative mechanism, such as Diels–Alder.^9^ Several examples of other chemistries that have been employed in CANs include esters,^10,11^ vinologous-urethanes,^12^ and imines.^13^ Imine vitrimers are of interest due to their facile synthesis and rapid reversibility at a moderate temperature (Figure S1).^14−16^

**Figure 1:**
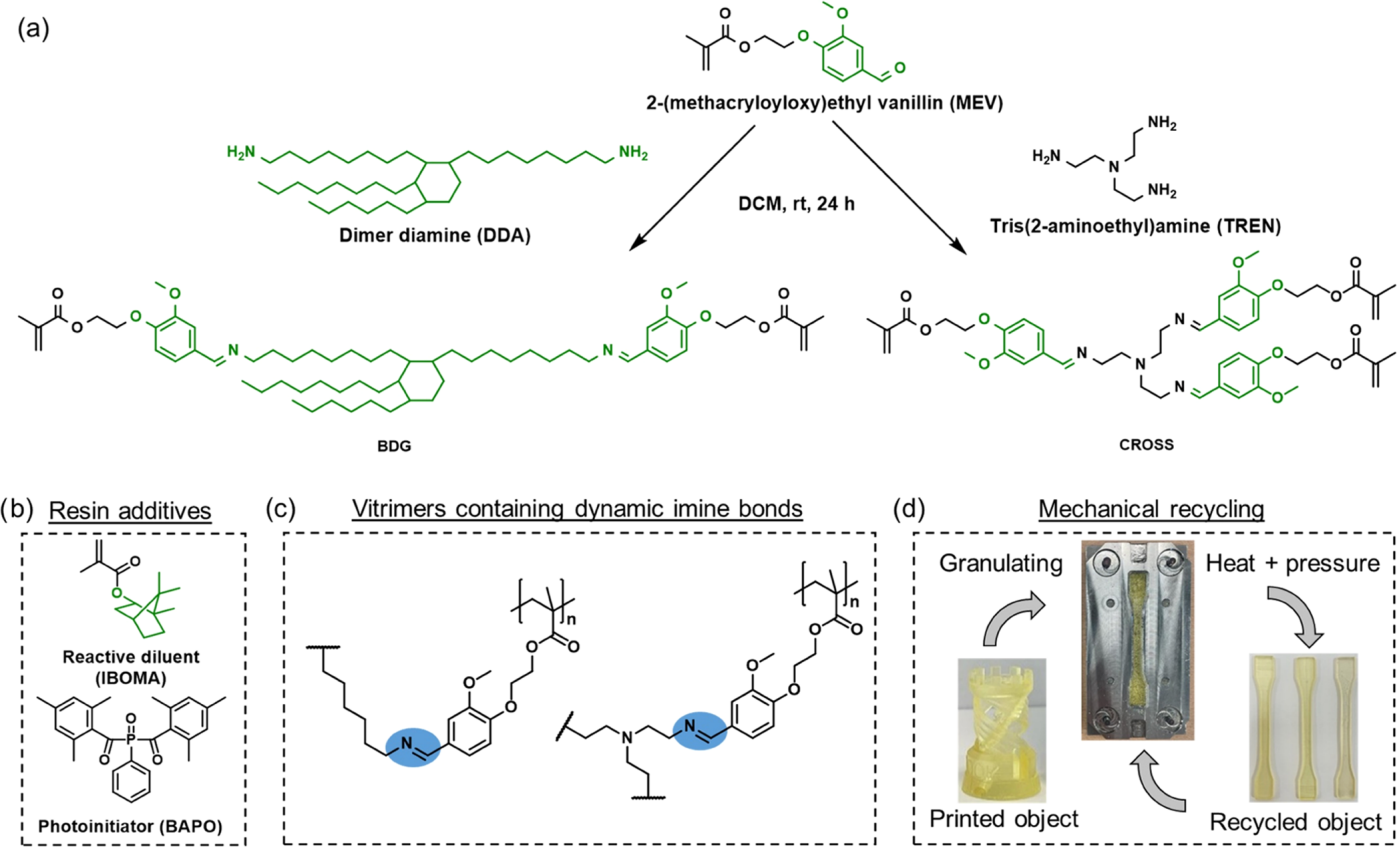
(a) Reaction scheme depicting the synthesis of building blocks BDG and CROSS. (b) Additives used in this work to prepare the resin formulations. (c) Schematic molecular structure of the cross-linked resins containing dynamic imine bonds. (d) Overview of the recycling process of the 3D-printed vitrimer resins.

Vanillin is a promising intermediate in the production of biobased imine vitrimers and is currently produced on an industrial scale from lignin.^17^ In addition to its capability of rapidly forming imines in the presence of primary amines, its phenol group allows for further modification into methacrylate functionality. These two aspects enable its use in DLP as part of a UV-curable resin. The use of vanillin methacrylate (VM) as a monomer^18^ or vanillin dimethacrylate as a building block^19,20^ in photocurable resins for 3D printing has been reported previously. In only a few cases, vanillin was reported as being part of the larger cross-linkable molecule containing reversible imine bonds. The Schiff-base formation between VM and a primary amine allows for the facile production of mono- or multifunctional monomers with various molecular structures simply by changing the amino functional molecule. For example, the reaction between fossil-derived Jeffamines^21^ or 4,4′-oxydianiline^22^ and VM was reported. The resulting resins were used in photopolymer formulations. Inspired by the wide variety of molecular structures and functionalities that biobased resources have to offer,^23,24^ we propose the use of the fully biobased Priamine 1075 as the amino functional molecule in combination with 2-(methacryloyloxy)ethyl vanillin (MEV)^25^ to obtain a difunctional photopolymer (BDG in Figure 1a). Priamine 1075 is a dimer diamine (DDA) based on fatty acids, bringing comparatively high flexibility to the resin. Furthermore, except for the methacrylic acid group, the building block can be fully obtained from biobased resources. In total, the building block BDG is 78% biobased.

To bring higher network density and imine concentration to the resin, the addition of the trifunctional alternative of DDA, namely, tris(2-aminoethyl)amine (TREN), was combined with MEV to prepare a trifunctional cross-linker (CROSS in Figure 1a). The generally high network density resulting from the proposed building blocks will bring some desirable properties, such as high mechanical rigidity and *T*_g_, while also retaining rapid imine exchange, promoting facile recycling of the vitrimer. The imine concentration in a vitrimer system is associated with the activation energy due to the dilution effect, resulting in more rapid network relaxation at a higher imine concentration.^26,27^ In contrast to the previously reported VM, we propose the use of a vanillin methacrylate monomer containing an ethoxy spacer between the methacrylate and phenol groups. The ethoxy group built in the monomer that follows from a two-step reaction, improves the hydrolytic stability of the monomer.^28,29^

The use of BDG is proposed as part of a 3D printing resin formulation containing biobased isobornyl methacrylate as the reactive diluent and a photoinitiator (Figure 1). The thermal and mechanical properties of the UV-cured and 3D-printed materials are evaluated. The introduction of dynamic covalent imine bonds as a part of the proposed BDG and CROSS building blocks will enable the rapid mechanical recyclability of printed objects. The resins reported herein can be mechanically recycled as a secondary raw material instead of the primary resinous material employed in the initial DLP process. Nonetheless, the reported resin formulations can contribute to a reduction in the amount of plastic waste.

## Experimental Section

### Materials

Tris(2-aminoethyl)amine (TREN, 96%), sodium hydroxide (>99%), magnesium sulfate (>99.5%), isobornyl methacrylate (IBOMA, technical grade), butylated hydroxytoluene (BHT, >99%), and phenylbis(2,4,6-trimethylbenzoyl)phospineoxide (BAPO, 97%) were all purchased from Sigma-Aldrich. 2-(Methacryloyloxy)ethyl vanillin (MEV, 98%) was custom-synthesized by Ecosynth (Belgium).^25^ Priamine 1075 was kindly provided by CRODA. All solvents were purchased from Biosolve.

The reference material Liqcreate Deep Blue was kindly provided by Liqcreate. BMPR-06 is a resin formulation based on previous research by NHL Stenden University, consisting of acrylated soybean oil and isobornyl methacrylate (IBOMA).^30^

### Synthesis of the Bifunctional Vanillin-Based Building Block Bridge (BDG)

To a 2 L three-neck round-bottom flask equipped with a mechanical overhead stirrer were added Priamine 1075 (159.06 g, 0.298 mol, 1 equiv), MEV (165.50 g, 0.626 mol, 2.1 equiv), and 1500 mL of DCM as the solvent. The reaction mixture was stirred at room temperature for 20 h under a nitrogen atmosphere. The mixture was filtered over a Buchner filter to remove insoluble byproducts, followed by concentrating the filtrate under reduced pressure at room temperature. After concentrating to half of the volume, butylated hydroxyl toluene (BHT) (2.9 g, 1 wt % relative to BDG) was added to the mixture, and the remaining DCM traces were removed under strong vacuum at room temperature. This resulted in a light yellow, viscous material with a yield of 87%. BDG was protected from light and stored at 4 °C. The general reaction scheme is shown in Figure 1a. ^1^H NMR (300 MHz, CDCl_3_): δ (ppm) = 0.76–1.80 (m, 66H, Aliphatic protons of DDA); 1.94 (s, 6H, C*H*_3_ methacrylate); 3.57 (t, 4H, C=N-C*H*_2_-); 3.91 (s, 6H, -O-C*H*_3_); 4.31 (t, 4H, (C=O)-O-C*H*_2_-C*H*_2_-); 4.53 (t, 4H, (C=O)-O-C*H*_2_-C*H*_2_-); 5.57 (s, 2H, C=C*H*_2_, methacrylate); 6.13 (s, 2H, C=C*H*_2_, methacrylate); 6.93 (d, 2H, C*H* aromatic); 7.10 (s, 2H, C*H* aromatic); 7.42 (s, 2H, C*H* aromatic); 8.16 (s, 2H, C*H*=N). ^13^C NMR (75 MHz, CDCl_3_): δ (ppm) = 14.1; 18.2; 22.7; 27.0; 27.4; 29.7; 31.0; 31.9; 49.4; 56.0; 61.7; 63.0; 67.1; 67.3; 72.7; 109.7; 113.5; 122.6; 126.0; 130.6; 136.0; 150.1; 150.2; 160.1; 167.2. The ^13^C NMR spectrum is shown in Figure S2. FTIR cm^–1^: 2922 (s), 1721 (s), 1643 (m), 1588 (m), 1513 (s). HRMS (ESI+) *m*/*z*: [M + H]^+^ Calcd for C_64_H_103_N_2_O_8_ 1027.7714; Found 1027.6901. The HRMS spectrum is shown in Figure S3. The UV–vis spectrum is shown in Figure S4.

### Synthesis of the Trifunctional Vanillin-Based Cross-Linker (CROSS)

To a 1 L three-neck round-bottom flask equipped with a mechanical overhead stirrer were added TREN (24.01 g, 0.164 mol, 1.2 equiv), MEV (108.75 g, 0.411 mol, 3.0 equiv), and 500 mL of DCM as the solvent. The reaction mixture was stirred at room temperature for 20 h under a nitrogen atmosphere. The mixture was filtered over a Buchner filter to remove insoluble byproducts, and the filtrate was transferred to a separating funnel. The organic layer was washed three times with 150 mL of 1 M NaOH solution to remove unreacted TREN, followed by washing with brine. The organic layer was dried over anhydrous MgSO_4_. After filtration, BHT (1.19 g, 1 wt % relative to CROSS) was added, and the solvent was removed under strong vacuum at room temperature. A yellow highly viscous material was obtained with a yield of 76%. CROSS was protected from light and stored at 4 °C. The general reaction scheme is shown in Figure 1a. ^1^H NMR (300 MHz, CDCl_3_): δ (ppm) = 1.90 (s, 9H, C*H*_3_ methacrylate); 2.90 (t, 9H, C=N-CH_2_-C*H*_2_-); 3.65 (t, 6H, C=N-C*H*_2_-); 3.83 (s, 9H, O-C*H*_3_); 4.27; (t, 6H, (C=O)-O-C*H*_2_-C*H*_2_-); 4.49 (t, 6H, (C=O)-O-C*H*_2_-C*H*_2_-); 5.54 (t, 3H, C=C*H*_2_, methacrylate); 6.10 (s, 3H, C=C*H*_2_, methacrylate); 6.86 (d, 3H, C*H* aromatic); 6.94 (dd, 3H, C*H* aromatic); 8.05 (s, 3H, C*H*=N). ^13^C NMR (75 MHz, CDCl_3_) δ (ppm) = 18.3; 56.0; 56.1; 60.1; 63.0; 67.2; 109.7; 113.3; 122.7; 126.2; 130.5; 136.0; 150.1; 150.3; 161.3; 167.3. The ^13^C NMR spectrum is shown in Figure S5. FTIR cm^–1^: 2927 (b), 1714 (s), 1641 (m), 1583 (m), 1511 (s). HRMS (ESI+) *m*/*z*: [M + H]^+^ Calcd for C_48_H_61_N_4_O_12_ 885.4286; Found 885.3420. The HRMS spectrum is shown in Figure S6. The UV–vis spectrum is shown in Figure S4.

### Preparation of the Resin Formulations

Formulations were prepared with different ratios of BDG, CROSS, and IBOMA, as displayed in Table 1. The resin formulations were named by the weight fraction of CROSS present in the mixture and contained a total biobased content of 73–75%. The formulations for 3D printing were mixed in a 250 mL polypropylene cylinder, and the total amount required for printing was approximately 100 g. First, BAPO was dissolved in IBOMA, followed by the addition of the building blocks (Figure 1b). The formulations were mixed using a magnetic stirrer (200 RPM) at room temperature for at least 12 h.

**Table 1:** Composition of the Evaluated Resin Formulations All Containing 1 wt % BAPO Photoinitiator Relative to the Total Resin Formulation

formulation	CROSS	BDG	IBOMA	biobased content^a^
	(wt %)	(wt %)	(wt %)	(%)
CROSS-0	0	80	20	75
CROSS-5	5	75	20	74
CROSS-10	10	70	20	73

a Calculated from the resin composition, taking into account 1% of non-biobased BAPO, and the biobased content of BDG (78%), CROSS (51%), and IBOMA (69%).

### Preparation of Cast UV-Cured Freestanding Films

Freestanding films were prepared by casting the resin formulation in a circular PTFE dish with a diameter of 75 mm. The film was cured in a Formlabs Form Cure UV curing oven containing a 405 nm LED UV source of 39 W (LED radiant is 9.1 W) at 60 °C. The film was cured for a total of 30 min and rotated after 15 min to obtain homogeneous curing on each side. After UV curing, the films were transferred to a washing bath containing isopropyl alcohol (IPA) to remove any unreacted resin on the surface. The final film thickness was between 0.62 and 0.74 mm.

### 3D Printing

3D printing was performed using a Phrozen Sonic Mini 4K 3D (DLP) printer with a 405 nm ParaLED Matrix 2.0 light source with an irradiance of 2.3 mW/cm^2^. The print models were prepared using Chitubox V1.9.2, and all of the samples were printed with a layer height of 100 μm and the corresponding exposure time. Tensile bars (ISO 527-2-1 BA), impact bars (ISO 179-1), Rook Towers (thermal resistance test), and stress relaxation plates were printed. After printing, the samples were washed in IPA for 20 min (Formlabs Form Wash), air-dried for 20 min, and then postcured for 30 min at 60 °C (Formlabs Form Cure, 405 nm, LED radiant is 9.1 W).

Before the samples were printed, a working curve was prepared for each formulation. This was needed to determine the exposure time of the formulation to achieve a layer thickness of 100 μm. To prepare the working curve, three microscope slides were placed on the bottom of the empty and clean resin tank of the 3D printer. An amount of the formulation to cover the entire slide was applied with a pipette and was then exposed to different irradiation times from 4 to 22 s with a 2 s time interval. The thickness of each layer was measured to create a working curve, which represents the exposure time corresponding to the layer thickness. The exposure time corresponding to 150 μm was used as an input value to ensure that the printer prints a layer thickness of 100 μm. The exposure times were 16.0 s for CROSS-0, 11.7 s for CROSS-5, and 11.4 s for CROSS-10. From the slope of the working cure (Figure S11), the depth of penetration (*D*_p_) was determined to be 362 μm for CROSS-0, 279 μm for CROSS-5, and 404 μm for CROSS-10. The critical energy (*E*_c_) which is required for printing is calculated to be 23.8 mJ/cm^2^ for CROSS-0, 15.1 mJ/cm^2^ for CROSS-5, and 17.5 mJ/cm^2^ for CROSS-10. The general structure of the UV-cured building blocks, highlighting the dynamic imine groups, is shown in Figure 1c.

### Recycling of the 3D-Printed Vitrimer Resins

To demonstrate the recyclability of the 3D-printed vitrimer resins, the specimens were ground into a fine powder with an electric coffee grinder. One gram of the ground material was transferred into a dogbone-shaped mold with dimensions according to ISO 527-2-1BA. The resin was then pressed for 5 min at 150 °C and a pressure of 40 kN using a LabEcon 600 series by Fontijne Presses. This recycling step was repeated three times, each time measuring the tensile properties (Figure 1d).

### Characterization

#### Nuclear Magnetic Resonance (NMR) Spectroscopy

Structural characterization of the synthesized vanillin-based building blocks was performed by ^1^H NMR spectroscopy. The spectra were recorded on a Bruker Avance III HD Nanobay 300 MHz apparatus at 298 K in CDCl_3_ using 16 scans. The building blocks were dissolved in CDCl_3_.

#### High-Resolution Mass Spectrometry (HRMS)

Mass spectrometry data were collected on a Waters Synapt G2-S instrument employing an electrospray ionization (ESI) source using the following conditions: capillary voltage = 3.0 kV, sampling cone voltage = 50 V, cone gas flow rate = 50 L/h, desolvation gas flow (L/Hr) = 600 L/h, nebulizer gas flow = 3 bar and source temperature = 150 °C. Data were acquired in positive ESI mode, with a scan rate of 1 s per scan. The samples were dissolved in a mixture of acetonitrile and water 9:1, containing 0.1% acetic acid.

#### UV–VIS

Absorption spectra were recorded in the range from 200 to 800 nm using a Jenway spectrophotometer Model 7205. The samples were dissolved in DCM. DCM was also used as the blanco measurement using a quartz cuvette.

#### Viscosity

The viscosity of the resin formulations was measured using an Anton Paar Physica MCR300 parallel-plate rheometer. The diameter of the plates was 50 mm, and the distance between the two geometries was 1 mm. Flow sweep measurements were performed at 20 °C with a shear rate from 1 to 100 s^–1^.

#### Dynamic Mechanical Analysis (DMA)

DMA was performed on the cast UV-cured films. Rectangular samples were cut from the film with a width of 2 mm and a thickness between 0.62 and 0.74 mm. The *T*_g_ of the cross-linked films was obtained by recording a temperature ramp from 25 to 250 °C using a heating rate of 3 °C/min. The temperature ramp was performed in tension mode on a DMA 1 STAR system (Mettler Toledo) with a frequency of 1.0 Hz and an amplitude of 1.0%.

#### Thermogravimetric Analysis (TGA)

The thermal stability of the cross-linked resins was analyzed with TGA on a TA Instruments Qseries, TGA Q500 with autosampler using a temperature ramp from 25 to 700 °C with a rate of 3 or 10 °C/min under nitrogen flow. Isothermal thermal stability was measured at a temperature of 200 °C for 10 or 60 min under nitrogen flow.

#### Photorheology

Rheological measurements on the resin formulations in the presence of UV light were performed on an MCR 702 Multidrive rheometer from Anton Paar using a parallel-plate geometry. The bottom geometry consisted of a quartz glass plate. The top plate had a diameter of 15 mm. A time sweep was measured with a frequency of 10 rad/s and a strain of 1% while maintaining a gap of 1000 μm. During the measurement, the sample was illuminated from the bottom using a Dymax BlueWave 75 UV probe with a short arc bulb of 75 W. The distance between the probe and the sample was approximately 1 cm.

#### Tensile Test

For the mechanical properties of the 3D-printed parts, tensile bars with a length of 75 mm, a width of 5 mm, and a thickness of 2 mm according to ISO 527-2-1BA were printed. Tensile bars from the different recycling steps were prepared by using a mold with the same dimensions according to ISO 527-2-1BA. Tensile tests were performed on a Zwick UPM 14740 ZMART. PRO with a 5 kN load cell and a crosshead speed of 5 mm/min at room temperature. All of the samples were placed in a climate cabinet 24 h in advance at 23 °C and a relative humidity of 50%. A minimum of five specimens per resin formulation were measured.

#### Fourier Transform Infrared (FTIR) Spectroscopy

Characterization of the chemical structure was performed using a Bruker Vertex 70 FTIR spectrophotometer with an ATR accessory. Sixteen scans were performed in the range of 4000 to 400 cm^–1^ with a resolution of 4 cm^–1^. An FTIR spectrum of the 3D-printed resins was recorded before printing, after printing, and after each reprocessing cycle. Spectra were normalized to the peak at 1510 cm^–1^.

#### Thermal Resistance

The thermal stability of the 3D-printed specimens was demonstrated by placing a printed Rook Tower of each formulation in an oven at 200 °C for 10 min and evaluating the structural integrity visually. The specimen should show no visible deformation to exhibit good thermal resistance.

#### Stress Relaxation

The stress relaxation of the 3D-printed specimens was evaluated on an Anton Paar Physica MCR 302e with an 8 mm parallel-plate configuration. All of the experiments were performed under a constant strain of 1% with an axial force of 1 N for 1000 s, where the characteristic relaxation time τ* was determined at 1/*e* of the initial stress. Each formulation was tested at different temperatures (60, 90, 120, 150, and 180 °C). All of the samples were 3D-printed and had a thickness of 0.8 mm and a diameter of 12 mm.

#### Gel Content

The gel content of the cross-linked formulations was determined using a Soxhlet extractor with tetrahydrofuran (THF) as the solvent for 48 h. After extraction, the samples were dried in a vacuum oven at 40 °C overnight. The samples were weighed before and after extraction. The gel content was determined according to eq 1.
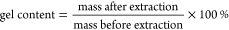
1

## Results and Discussion

### Synthesis of BDG and CROSS

**Figure 2:**
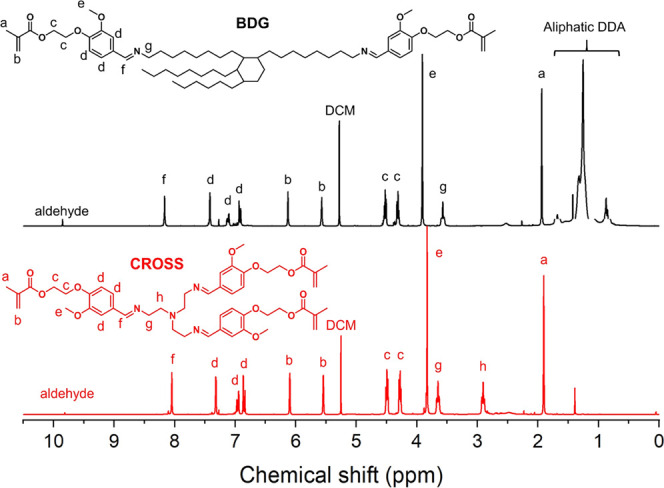
Overlay of the assigned ^1^H NMR spectra of the building blocks recorded in CDCl_3_. Black, BDG; red, CROSS.

The building blocks BDG and CROSS were both obtained in good yields of 87 and 76%, respectively. Due to the high viscosity of the purified compounds, it was challenging to completely remove all traces of DCM in the final products, as observed in the ^1^H NMR spectra at 5.30 ppm (Figure 2). However, the proposed structures correspond well with the obtained spectra (Figure 2). The resonance at 8.1 ppm corresponds to the imine proton and confirms the presence of dynamic imine bonds between MEV and Priamine 1075 or TREN. A small amount of free aldehyde (resonance appears at 9.85 ppm), which corresponds to the MEV molecule, was present in each building block. A slight excess of free aldehyde was desired to avoid the presence of free amine, which can cause cross-linking reactions through aza-Michael addition reactions with the methacrylate groups of MEV. Samples of building blocks that contained residual amounts of free amine according to ^1^H NMR spectroscopy gelled during storage despite the addition of 1% BHT to prevent radical reactions on the methacrylate groups. Therefore, gel formation suggests coupling of the building blocks via Michael addition reactions. Despite being poor Michael acceptors, methacrylates can still react slowly with primary amines to form the Michael adduct.^31^ This was confirmed in a model reaction between hexyl amine and IBOMA. An equimolar mixture of hexyl amine and IBOMA was reacted at room temperature, and the internal standard (IS) trioxane was added to follow the disappearance of the methacrylate protons. The ^1^H NMR spectra in Figure S7 show the slow disappearance of methacrylate groups and the formation of the aza-Michael adduct. Samples of the building blocks containing a slight excess of aldehyde and no free amine did not gel during storage. During 3D printing, excess MEV will participate in the reaction, leaving no unreacted monomers in the resin.

### Properties of the UV-Cured Resins

Resin formulations were prepared using IBOMA as the reactive diluent, various amounts of BDG and CROSS as the difunctional and trifunctional monomers, respectively, and BAPO as the photoinitiator. The compositions are listed in Table 1. Initial characterization by photorheology confirms the rapid cross-linking under UV light. A cross-linked network was obtained within 1 s after switching the light on (Figure S8). It must be noted that the intensity of the lamp used in the photorheology measurements is orders of magnitude higher than what is illuminated in the 3D printer.

**Figure 3:**
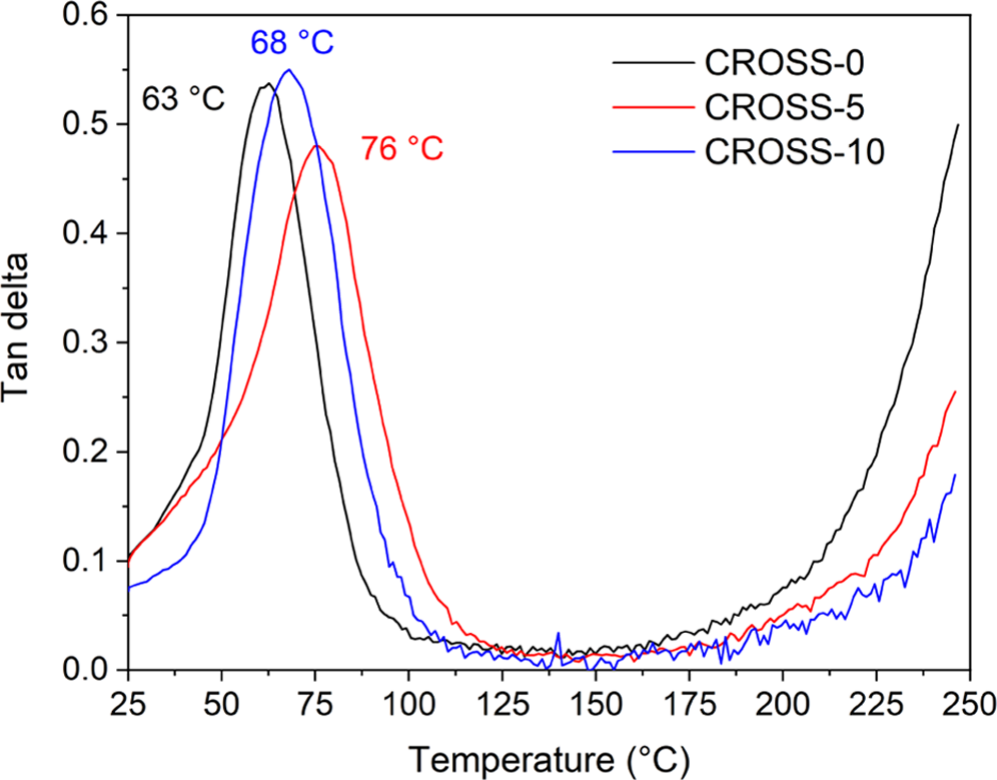
DMA results of the cross-linked resins CROSS-0 (black), CROSS-5 (red), and CROSS-10 (blue), displaying the tan delta for each vitrimer.

For further characterization of the resin formulations CROSS-0, CROSS-5, and CROSS-10, cross-linked freestanding films were prepared in a UV oven after casting the resin in a PTFE dish. All of the cross-linked resins exhibited a high gel content of between 97.8 and 98.1%. The effect of the amount of trifunctional CROSS on the *T*_g_ of the cross-linked resin was investigated using DMA (Figures 3 and S9). While it is expected that rigid and trifunctional CROSS would significantly affect the thermal properties of the cross-linked resins, only a small effect on *T*_g_ was observed. Cross-linked resins with a *T*_g_ between 63 and 76 °C were obtained. It was reported in a previous study that by increasing the amount of cross-linker beyond a certain fraction of the total resin, only a marginal effect on the molecular weight between cross-links and *T*_g_ of the polymer was observed.^32^ Since the majority of the resin herein (80 wt %) consists of di- or trifunctional methacrylate, a similar effect is observed when part of the BDG is replaced by CROSS.

### 3D Printing

The pure building blocks BDG and CROSS exhibit a viscosity that is too high for 3D printing. Therefore, the building blocks were formulated in a resin containing the reactive diluent IBOMA. The addition of 20 wt % IBOMA resulted in a decrease in the viscosity. Samples containing more of the highly viscous CROSS increased in viscosity but overall remained below 7 Pa·s, which is within the capabilities of the 3D printer. The graph depicting the viscosity as a function of the shear rate is shown in Figure S10.

Using a working curve, the exposure time needed for each resin to obtain a layer thickness of 100 μm was determined. The exposure time corresponding to 150 μm was used as an input value to ensure that the printer prints a layer thickness of 100 μm. The exposure time used for each resin is listed in Table S1. The working curves are shown in detail in Figure S11. Using the optimized exposure times, objects including tensile bars, impact bars, rheology disks, and Rook Towers were successfully printed. The printed materials were rigid and yellow in appearance, which is caused by the presence of imine bonds (Figure S12).

**Figure 4:**
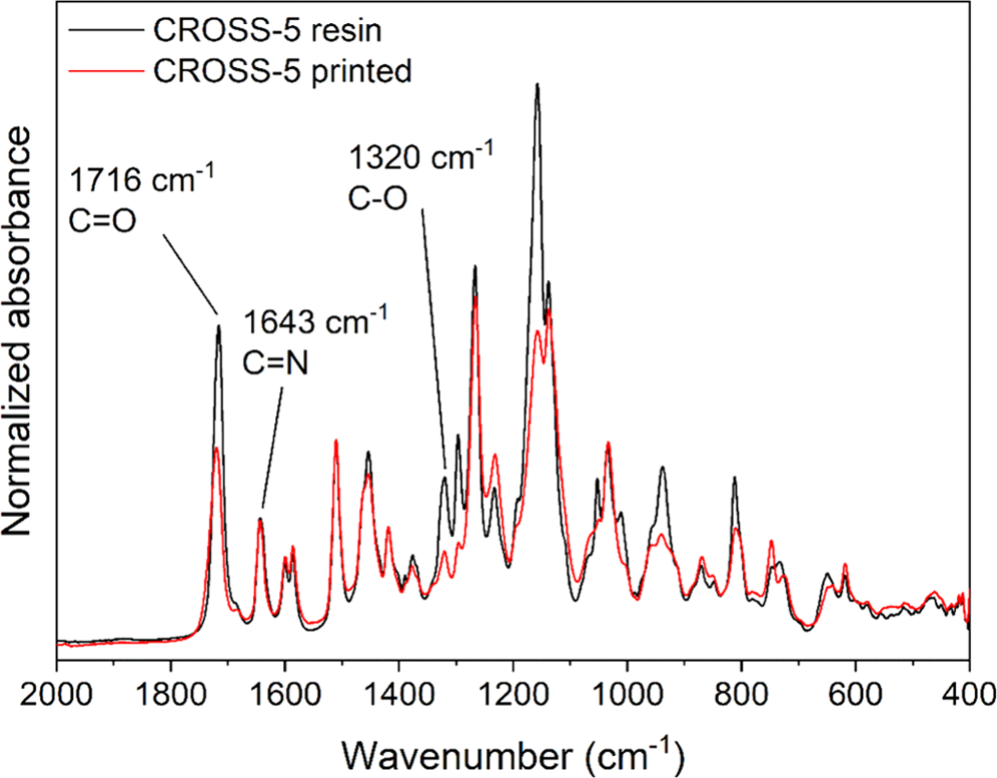
Overlay of the FTIR spectra of CROSS-5 before and after printing (spectra normalized at 1510 cm^–1^).

The UV curing was monitored by FTIR by observing the disappearance of the C–O vibration at 1320 cm^–1^ (Figure 4).^33^ After 3D printing and postcuring, the band at 1320 cm^–1^ was significantly reduced, indicating the conversion of methacrylate groups. The typically weak C=C band is not visible in the spectrum and possibly overlaps with the C=O band at 1716 cm^–1^ or with the imine signal present at 1643 cm^–1^.

**Figure 5:**
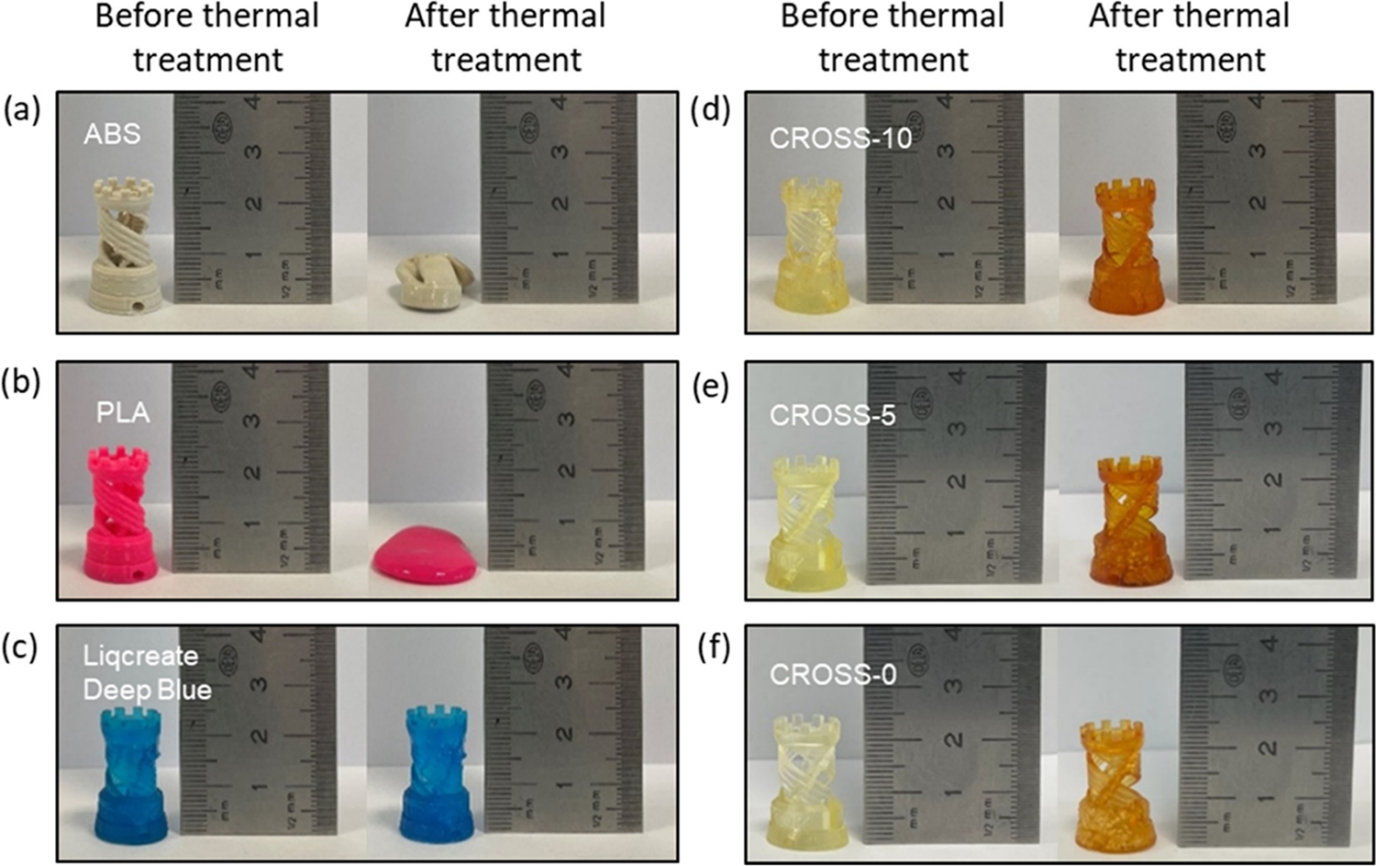
Photograph of the 3D-printed Rook Tower test specimens before and after thermal treatment at 200 °C for 10 min. The evaluated specimens are composed of thermoplastics ABS and PLA (a, b), thermoset Liqcreate Deep Blue (c), and vitrimers CROSS-0, CROSS-5, and CROSS-10 (d–f).

The 3D-printed Rook Towers prepared from the vitrimer resins were exposed to a thermal treatment of 200 °C for 10 min. The vitrimer test specimens were compared to those prepared from acrylonitrile butadiene styrene (ABS), polylactic acid (PLA), and Licreate Deep Blue (Figure 5a–c). The test specimens composed of thermoplastic ABS and PLA soften and melt under the evaluated test conditions, as expected. The specimens composed of thermoset and vitrimer material retain their structural integrity. CROSS-10, CROSS-5, and CROSS-0 show a slight deformation after the thermal resistance test (Figure 5d–f). At temperatures far above the *T*_g_, softening and sagging of the structure can occur. Nonetheless, the network consisting of dynamic cross-links is robust enough to withstand high-temperature conditions for a short period. Slight deformations can also be caused by tension present in the network caused by rapid curing during 3D printing. Another observation is the significant discoloration after thermal treatment of the vitrimer resins. The untreated specimens are pale yellow in appearance, whereas after the thermal treatment, a discoloration is observed.

Quantitative thermal stability was assessed using thermogravimetric analysis under nitrogen atmosphere (Figure S13). Each cross-linked resin showed a very similar degradation profile with a *T*_5%_ degradation at 312 °C. The printed parts were subjected to an isothermal heating step at 200 °C for 10 and 60 min (Figures S13a,b and S14). A slight discoloration was again observed afterward. However, a mass loss of maximum 2.4% was observed after 60 min, which could be attributed to moisture or residual solvent.

### Recycling

**Figure 6:**
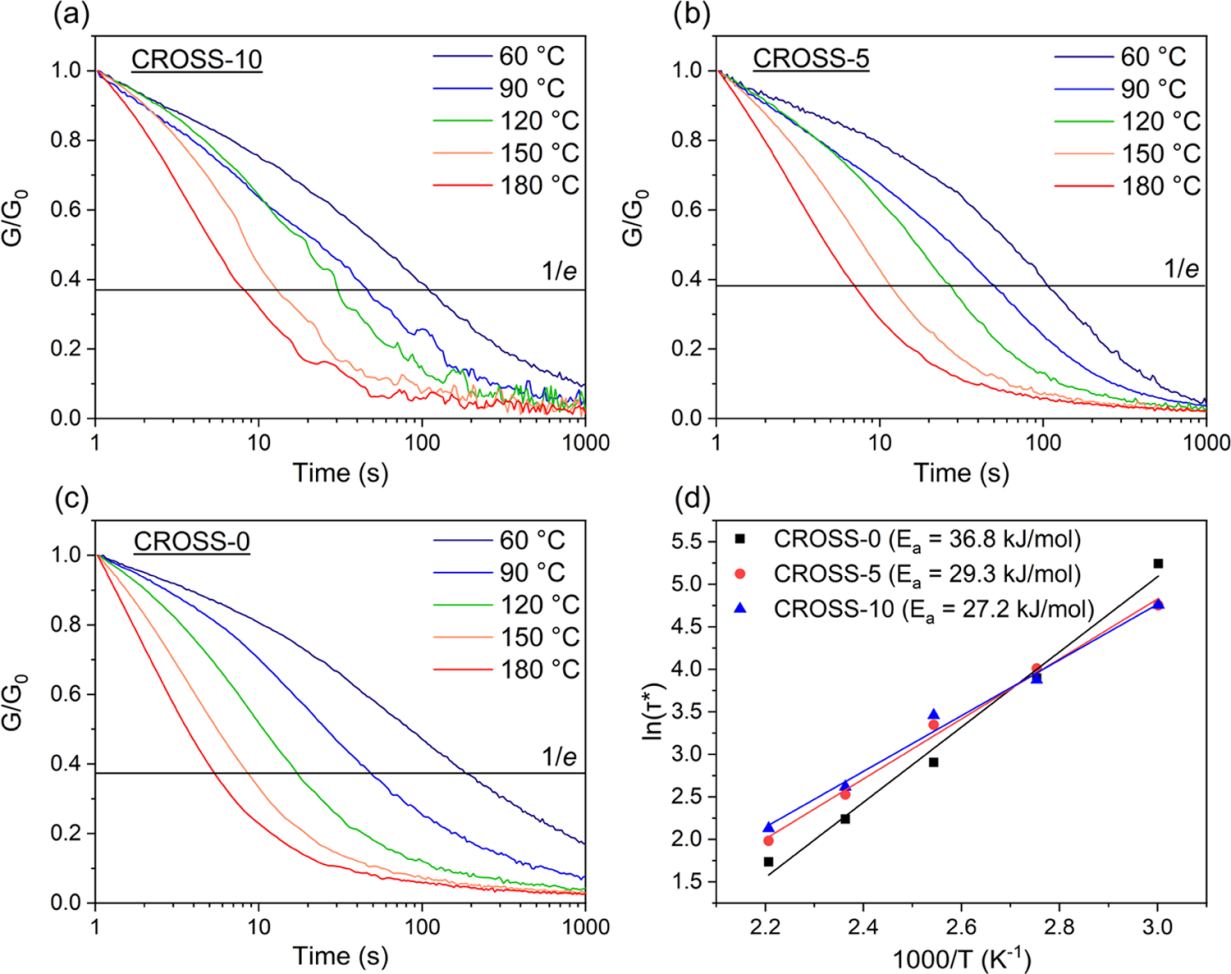
Normalized stress relaxation curves of (a) CROSS-10, (b) CROSS-5 and (c) CROSS-0. (d) Arrhenius plot ln(τ*) vs 1000/*T* (K^–1^) and linear fit to extract the activation energy (*E*_a_).

Due to the presence of imine bonds in the vanillin-based building blocks BDG and CROSS, the networks obtained display dynamic exchange of cross-links at elevated temperatures. This behavior can be quantified using rheological stress relaxation experiments. The 3D-printed samples were subjected to a constant strain while measuring the relaxation modulus over time. The characteristic relaxation time τ* is measured as the time needed to relax to 1/*e* (∼0.37) of the initial stress. The values are fitted in an Arrhenius plot to obtain the activation energy (*E*_a_). The results are shown in Figure 6.

All of the 3D-printed vitrimers show thermally activated relaxation of the network at temperatures between 60 and 180 °C. While the addition of CROSS did not significantly affect the *T*_g_ of the cross-linked polymer, a significant effect on the *E*_a_ was observed. The obtained values for *E*_a_ are 36.8 kJ/mol for CROSS-0, 29.3 kJ/mol for CROSS-5, and 27.2 kJ/mol for CROSS-10. The differences in *E*_a_ between the samples are related to the network density, with CROSS contributing to a higher network density relative to BDG. A higher network density means a higher probability of two imine bonds meeting for a metathesis exchange reaction, leading to a lower *E*_a_.^26,27^ In the vitrimer reported here, metathesis reactions are expected due to the slight excess of aldehyde groups in the building blocks BDG and CROSS, as mentioned previously. The values for *E*_a_ obtained in this work are comparatively low for imine vitrimers,^34^ suggesting a high imine concentration and rapid network relaxation.

The stress relaxation curves of the reference resins Liqreate Deep Blue and BMPR-06 based on permanent networks are shown in Figures S15 and S16. The networks were unable to relax due to the inherent irreversibility of the cross-links. This behavior is in contrast to the vitrimers presented here, which are able to relax fully after 1000 s at elevated temperatures.

**Figure 7:**
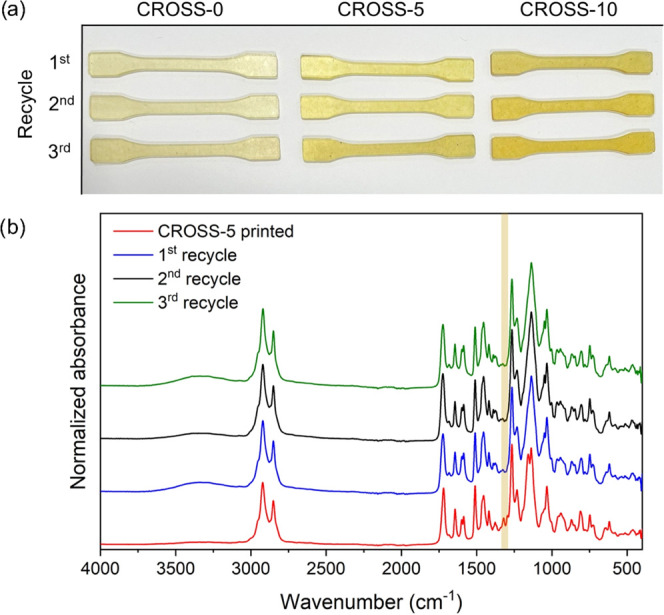
(a) Photograph of the recycled tensile bar specimens. (b) Overlay of the FTIR spectra of CROSS-5 after each recycling step (spectra normalized at 1510 cm^–1^).

The dynamic exchange of the vitrimer cross-links enables recycling of the 3D-printed specimens at elevated temperature and pressure due to the relaxation of the network and macroscopic flow of the material. To demonstrate this, 3D-printed material was ground into a powder and reprocessed in a mold at 150 °C and a force of 40 kN. The samples could be rapidly reprocessed. After 5 min in the press, a homogeneous sample was obtained from the granulated material. The process of granulating and molding into tensile bar-shaped specimens was repeated three times for each resin (Figure 7a). The tensile properties were measured after each cycle. Through the recycling steps, the material retained its physical appearance, showing only a slight discoloration after the third cycle. As a proof of concept, a tensile bar was broken and repaired in the mold under the same conditions as the recycling method. Microscope images of the break area show complete healing of the material, indicating the potential for the application as self-healing material (Figure S17).

In the FTIR spectra recorded after printing and after each recycling step, a slight decrease is observed in the bands corresponding to the C–O vibration at 1320 cm^–1^ (Figure 7b) after the first recycling step. This would suggest further conversion of the methacrylate groups during the recycling step. Furthermore, the FTIR spectra show no major changes, suggesting that no chemical degradation occurred during grinding and hot-pressing.

**Figure 8:**
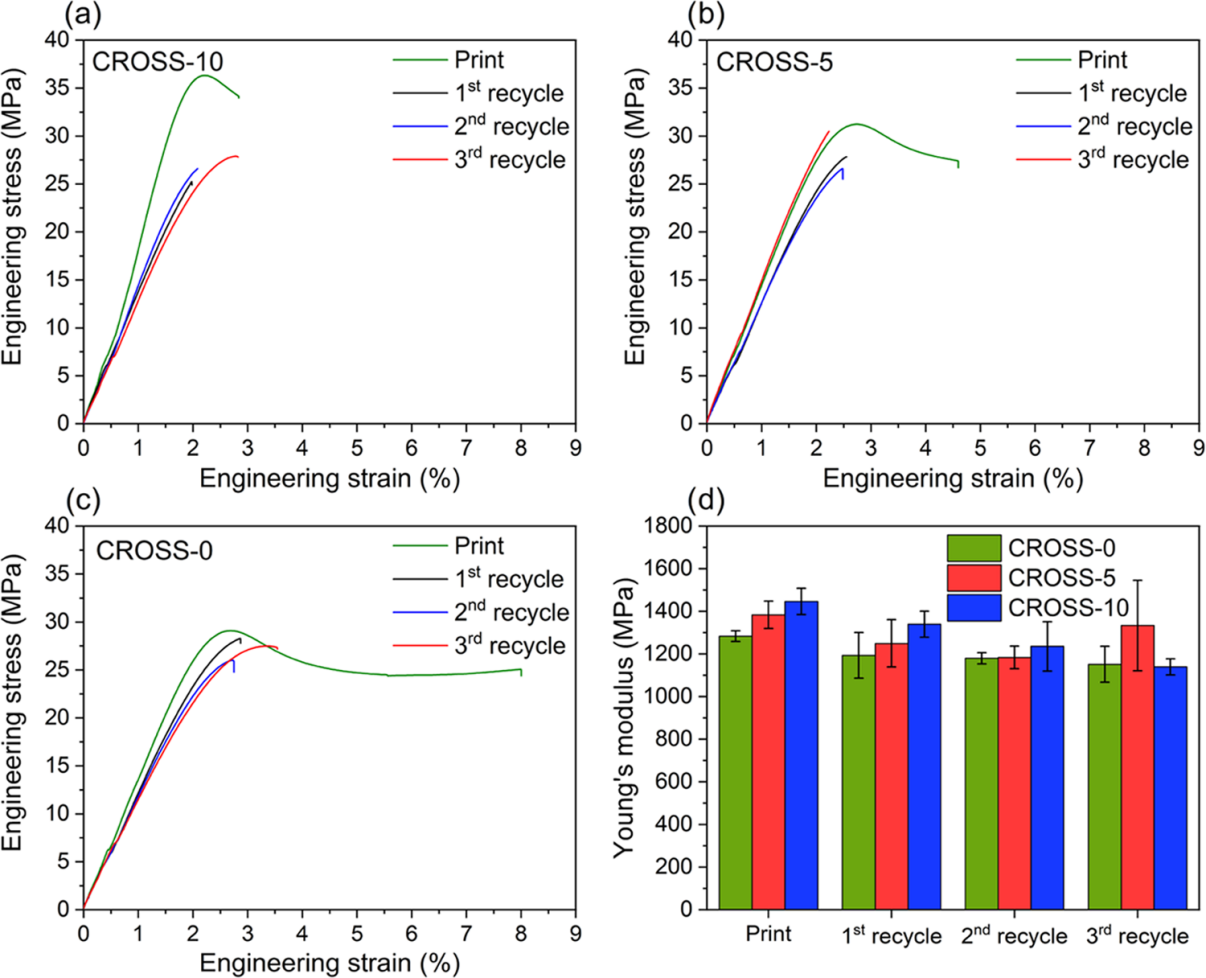
Tensile graphs of the printed tensile bars and recycled tensile bars of (a) CROSS-10, (b) CROSS-5, and (c) CROSS-0. (d) Bar graph depicting Young’s modulus after printing and after each recycling step for each of the resins.

Despite the high flexibility of BDG containing the dimerized fatty acid, rigid resins were obtained. Typically, cross-linked polymers with high rigidity are obtained when the *T*_g_ is above room temperature. In all cases, the printed tensile bar specimens showed yielding, which disappeared after recycling, resulting in lower values for strain at break (Figures 8a–c and S18a). This could be related to the observations made in FTIR, which showed the conversion of methacrylate groups after the first recycling step. Further conversion of methacrylate groups could increase the network density and reduce the strain at break of the recycled parts in contrast to the printed parts. When the printed specimens are compared, the formulations containing less CROSS showed a higher strain at break (8.5% for CROSS-0, 5.2% for CROSS-5, and 3.0 for CROSS-10) but a lower ultimate tensile strength. After recycling, the strain at break was reduced to approximately 2.5% for each formulation.

Resins with a larger amount of CROSS exhibited a slightly higher Young’s modulus and ultimate tensile strength (Figure 8). The strain at break remained largely the same throughout the recycling steps, with only CROSS-10 showing a significant difference between the print and recycled samples (Figure S18b). The Young’s modulus was maintained or decreased only slightly after several recycling steps (Figure 8d). This result shows that the mechanical rigidity of the vitrimers is retained after several recycling rounds, holding promise for future application as a recyclable 3D-printable material. The Young’s modulus of CROSS-5 increased slightly after the third recycling step, although the standard deviation also increased. Multiple recycles of individual samples could increase the potential for errors during reprocessing, which would explain the slight deviation in the mechanical properties.

## Conclusions

In this work, a biobased building block BDG consisting of dimer fatty diamine and vanillin was successfully synthesized and formulated to yield photopolymer resins for 3D printing. The resulting networks containing dynamic imine bonds could be rapidly thermally recycled in a mechanical fashion. The rheological relaxation of the network, which reflects macroscopic flow under elevated temperature and pressure, was accelerated with the addition of cross-linker CROSS containing a higher concentration of imine groups. Comparatively low activation energies of between 36.8 and 27.2 kJ/mol were obtained in rheological stress relaxation measurements. This was translated to rapid mechanical recyclability resulting in homogeneous parts after hot-pressing for only 5 min at 150 °C and 40 kN. While the addition of CROSS to the formulation did not significantly affect the *T*_g_ of the network, an influence on the mechanical properties was observed, resulting in more rigid networks.

The 3D-printed parts also showed good structural stability under isothermal conditions in contrast to specimens consisting of thermoplastic material. The improved thermal stability, as well as the rapid recyclability, highlights the benefit of the use of vitrimer materials in photopolymer 3D printing.

This work will contribute to enhancing the end-of-life perspective of 3D-printable thermoset resins in the transition toward the circular economy. The use of the 3D-printed material as a secondary raw material for an alternative processing method does implicate some deviation in the resourcing and final application of the recycled material in contrast to the primary use of the resin in the DLP process. Nonetheless, good mechanical properties can be maintained after recycling and a reduction of plastic waste can be expected with the use of vitrimer materials. Furthermore, the use of renewable materials such as dimer fatty diamine and vanillin is investigated in this work, which is essential to reduce the strong dependence on fossil feedstocks for commercial photopolymer resins.

## Supporting Information Available

Reaction scheme of MEV imine formation and imine exchange; effect of BHT on the stability of building block CROSS; photorheology graph of resin formulation CROSS-0; DMA curve of photocured resins; rheological flow sweep measurements of the formulations and synthesized building blocks; development of the 3D printing working curves of the investigated resin formulations; photograph of the 3D-printed objects; stress relaxation curves of the reference resin formulations.; bar graph with tensile results of the printed and recycled resin formulations (PDF)
