# Supplementary material for: Biobased Photopolymer Resin for 3D Printing Containing Dynamic Imine Bonds for Fast Reprocessability
Source: ACS Appl Mater Interfaces. 2023 May 23;15(22):27110–9. doi: 10.1021/acsami.3c01669 (PMC10251348; doi:10.1021/acsami.3c01669)
Supplement: Supplementary file 1 — am3c01669_si_001.pdf [file am3c01669_si_001.pdf]

Supporting Information

Jules Stouten [a], Geraldine H.M. Schnelting [b], Jerzy Hul [c], Nick Sijstermans [a], Kylian Janssen [b], Tinashe Darikwa [a], Chongnan Ye [d], Katja Loos [d], Vincent S.D. Voet [b]*, and Katrien V. Bernaerts [a]*

[a] Sustainable Polymer Synthesis Group, Aachen-Maastricht Institute for Biobased Materials (AMIBM), Faculty of Science and Engineering, Maastricht University, Brightlands Chemelot Campus, Urmonderbaan 22, 6167 RD Geleen, the Netherlands.

[b] NHL Stenden University of Applied Sciences, Professorship Circular Plastics, van Schaikweg 94, 7811 KL Emmen, The Netherlands

[c] Liqcreate, Texasdreef 7, 3665 CL Utrecht.

[d] Macromolecular Chemistry and New Polymeric Materials, Zernike Institute for Advanced Materials, University of Groningen, Nijenborgh 4, 9747 AG Groningen, The Netherlands

* To whom correspondence should be addressed.

Katrien Bernaerts: katrien.bernaerts@maastrichtuniversity.nl

Vincent Voet: vincent.voet@nhlstenden.com

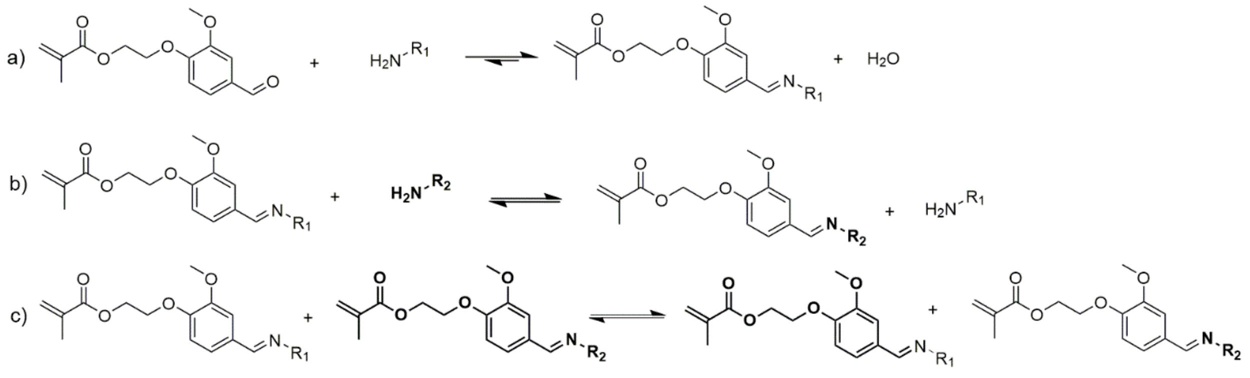

Figure S1. The thermoreversible reactions of MEV with a primary amine. a) Imine formation. b) Transamination. c) Metathesis.

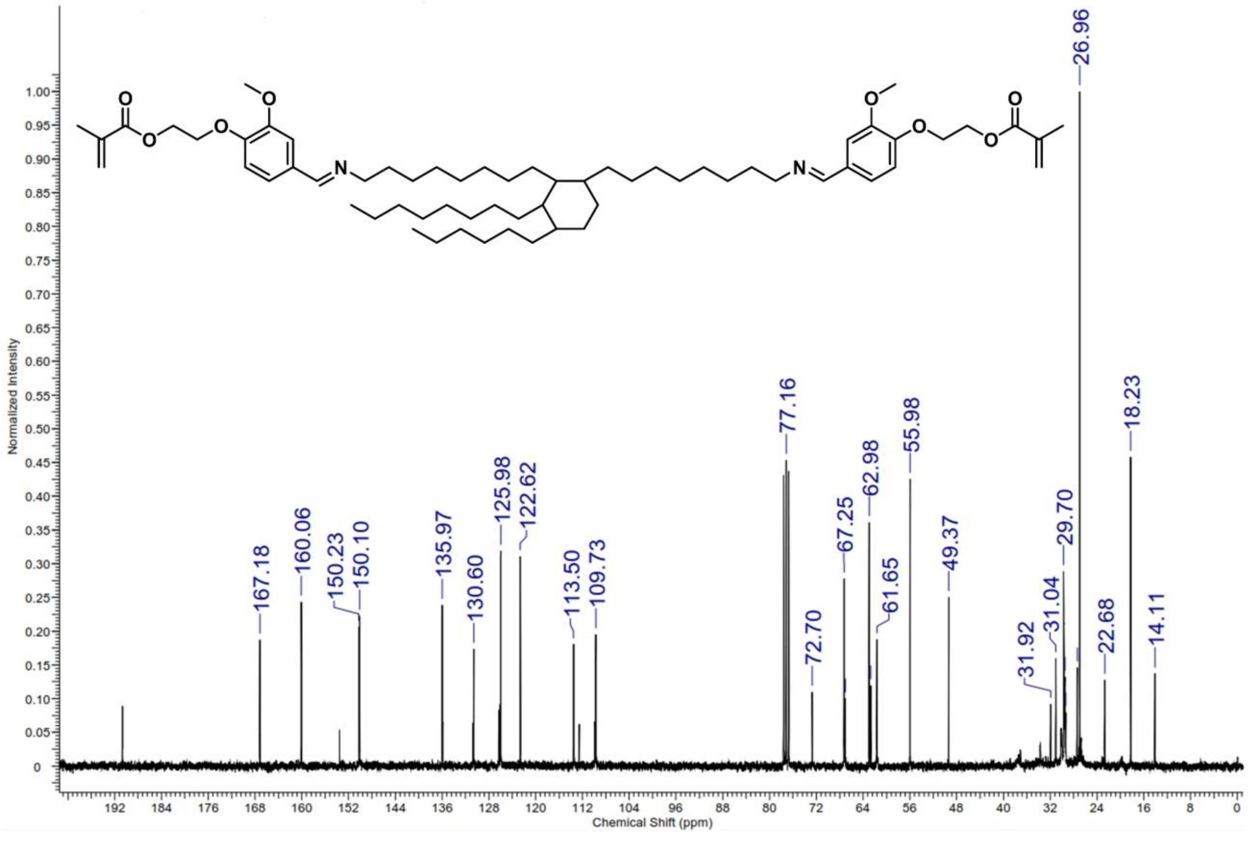

Figure S2. ^{13}C NMR spectrum of BDG.

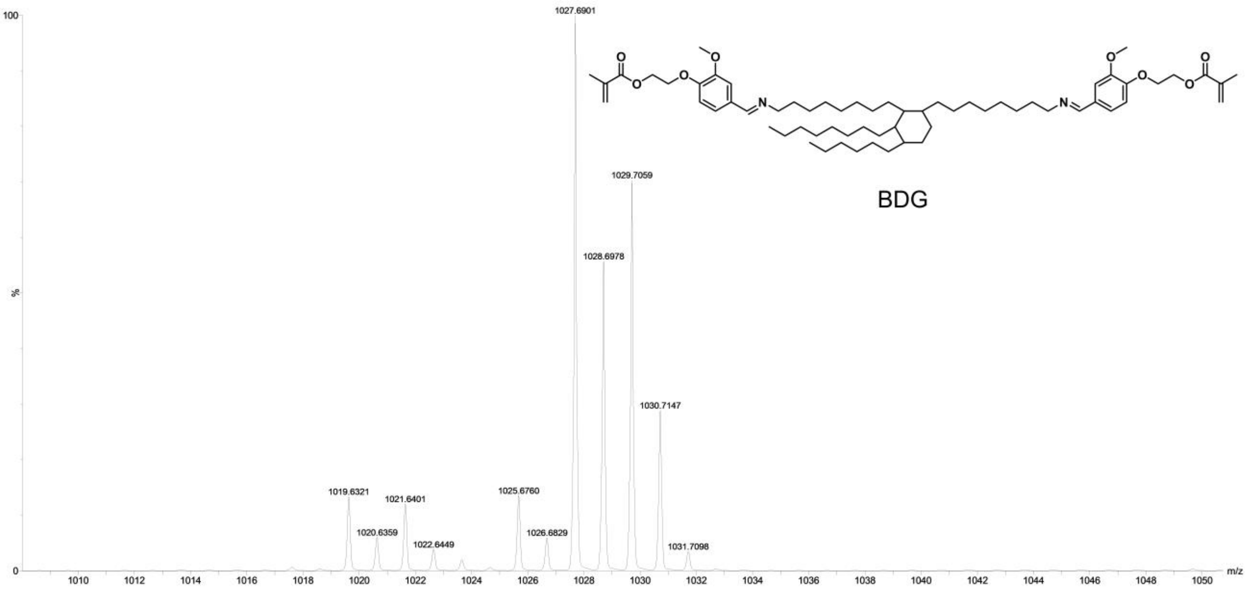

Figure S3. HRMS spectrum of BDG.

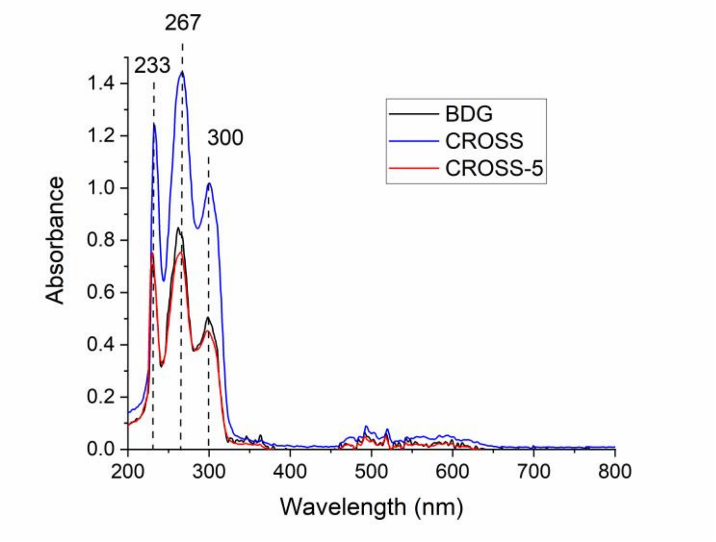

Figure S4. Overlay of the UV-VIS spectra of BDG, CROSS, and CROSS-5.

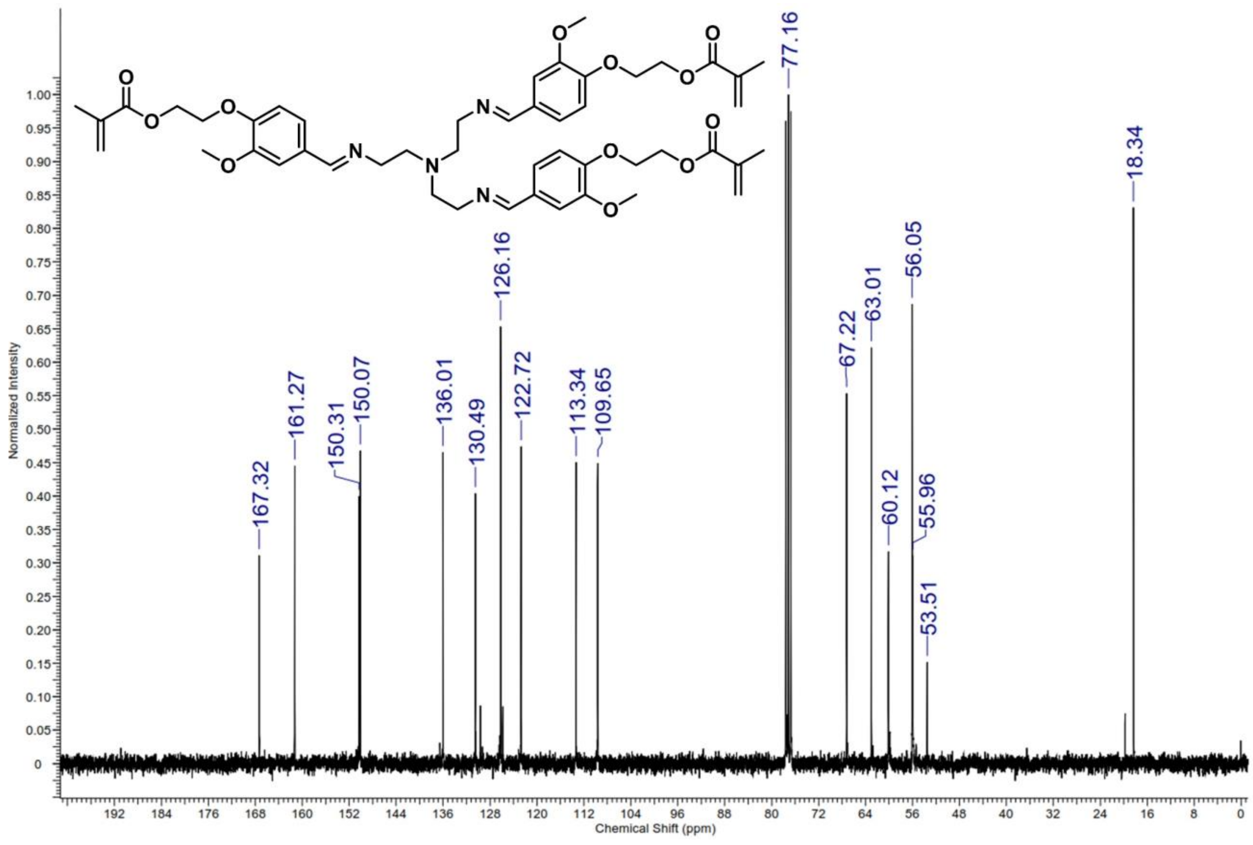

Figure S5. ^{13}C NMR spectrum of CROSS.

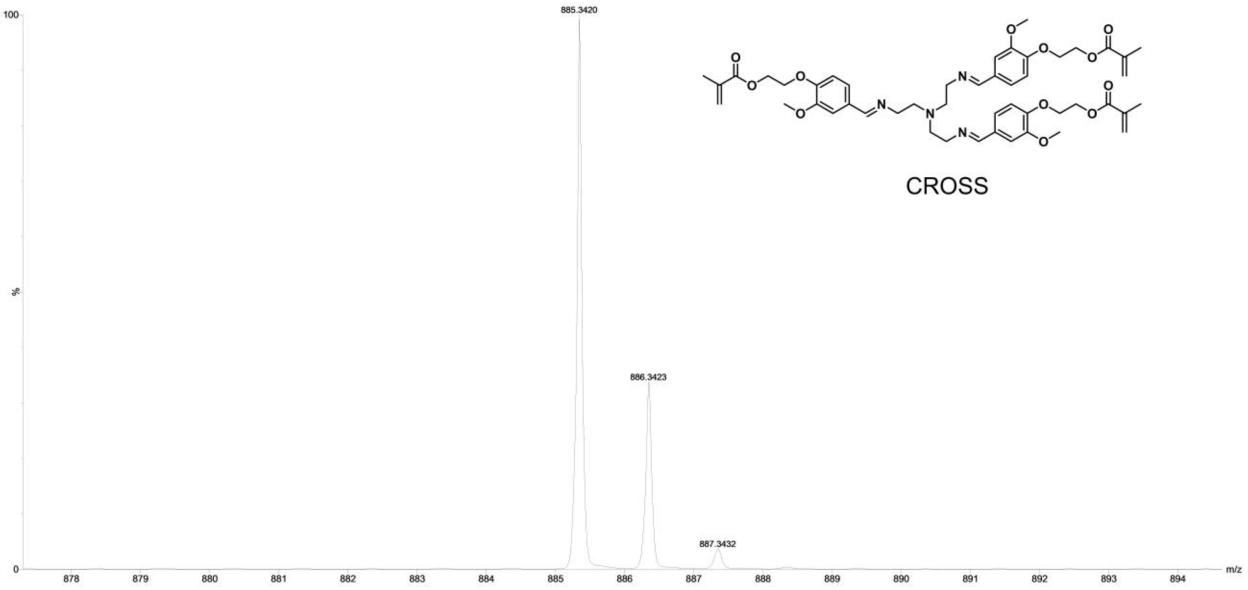

Figure S6. HRMS spectrum of CROSS.

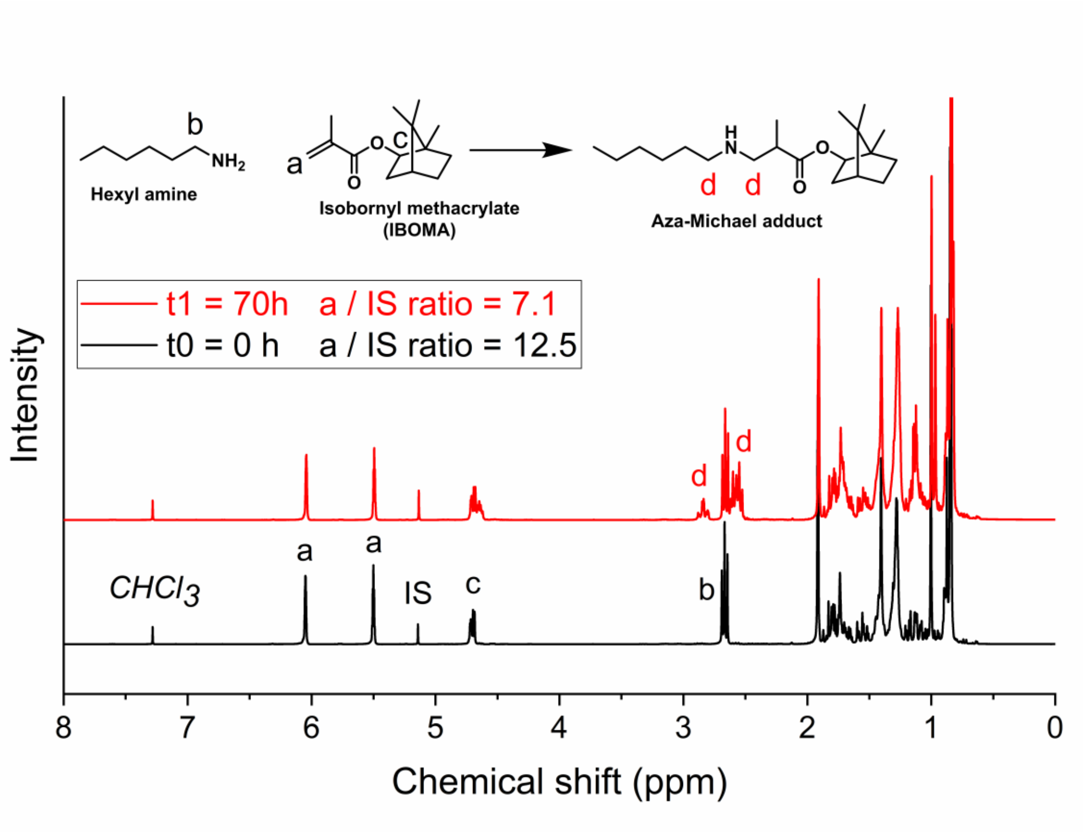

Figure S7. Overlay of the ^{1}H NMR spectra of reaction aliquots from the aza-Michael addition between hexyl amine and IBOMA after 0 and 70 hours.

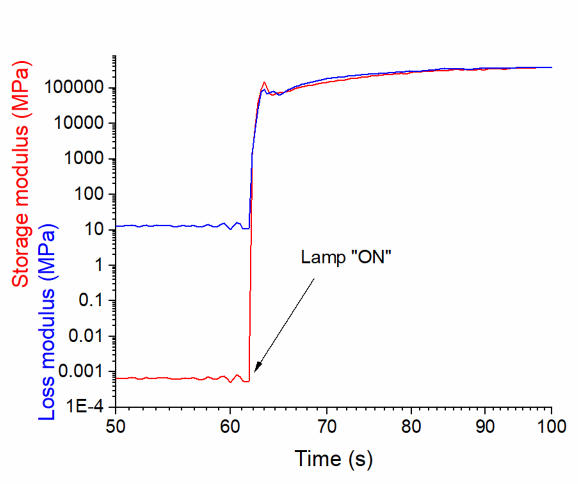

Figure S8. Photo-rheology graph of CROSS-0.

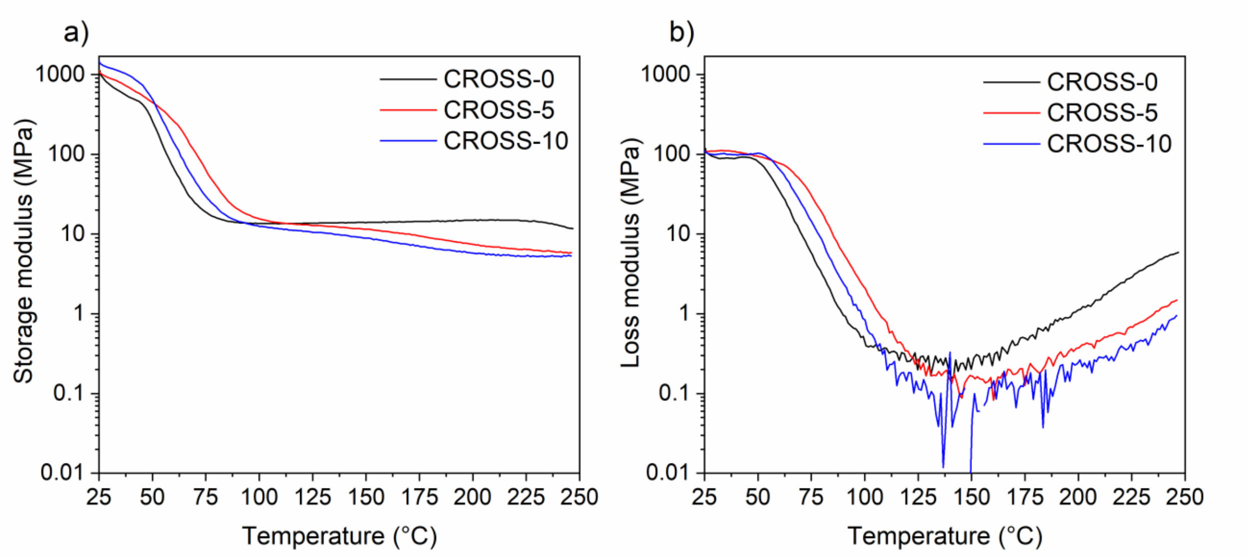

Figure S9. a) Storage and b) loss moduli obtained from the DMA scans of vitrimers CROSS-0, CROSS-5, and CROSS-10.

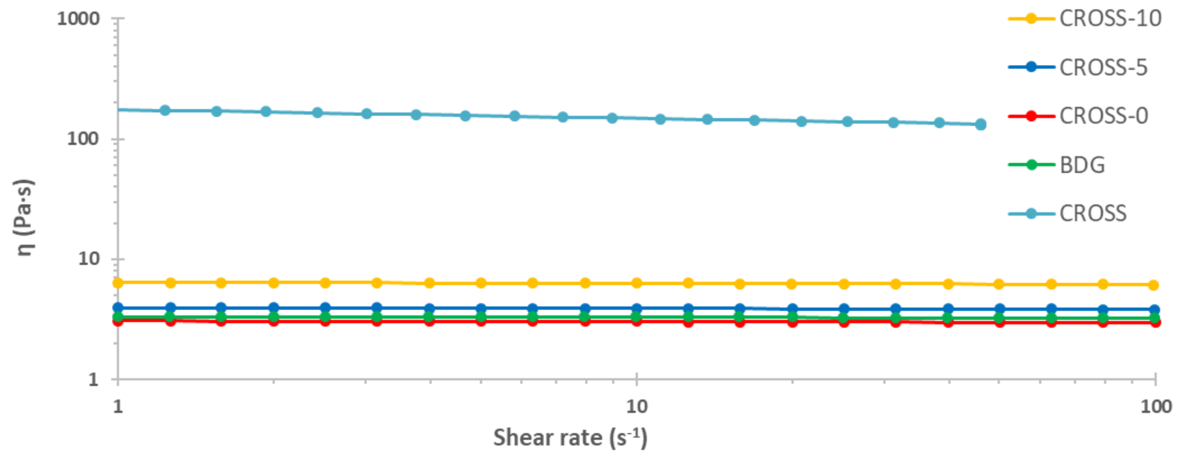

Figure S10. Viscosity as a function of the shear rate in a flow sweep measurement of the building blocks BDG and CROSS, and the formulations CROSS-0, CROSS-5, and CROSS-10.

Table S1. The UV exposure times and corresponding layer thickness for resins CROSS-0, CROSS-5, and CROSS-10.

**Layer thickness (µm)**	**Exposure time for CROSS-10 (s)**	**Exposure time for CROSS-5 (s)**	**Exposure time for CROSS-0 (s)**
100	9.7	9.3	13.7
150	11.4	11.7	16.0
200	13.1	14.2	18.2

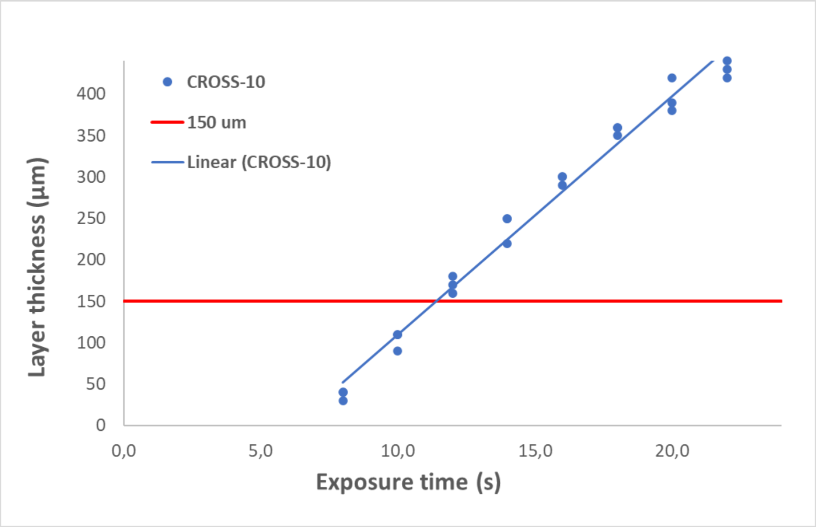

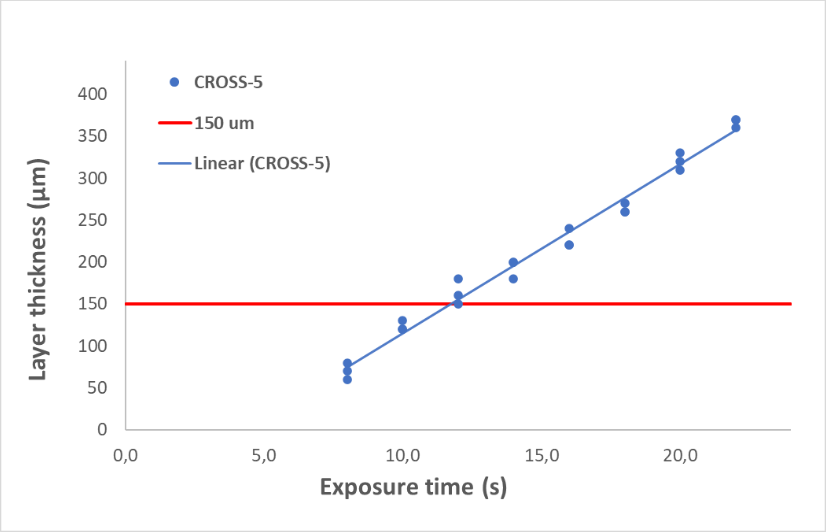

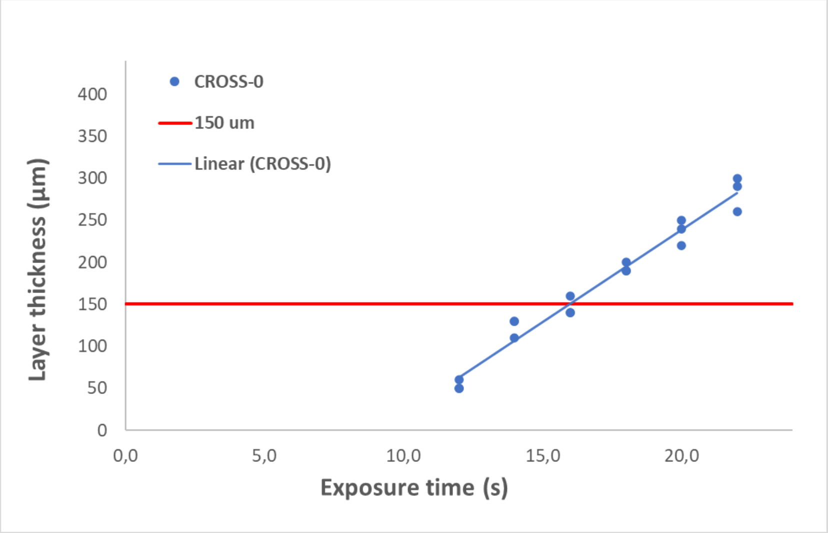

Figure S11. Exposure times determined by working curves in triplo for CROSS-10, CROSS-5 and CROSS-0

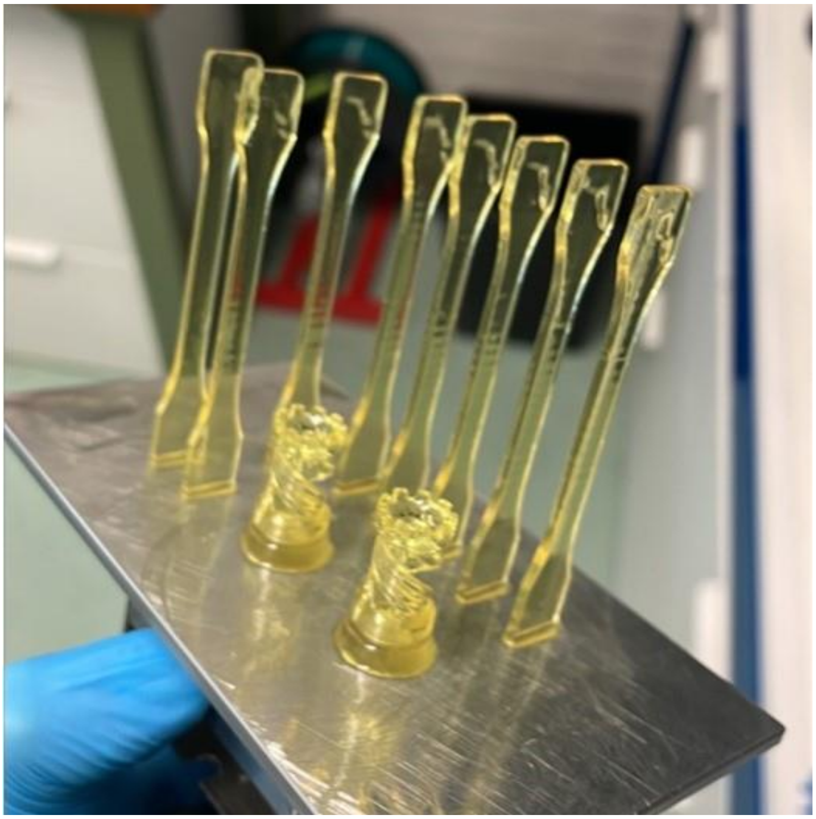

Figure S12. Photograph of the printed tensile bars and Rook Towers from resin CROSS-5.

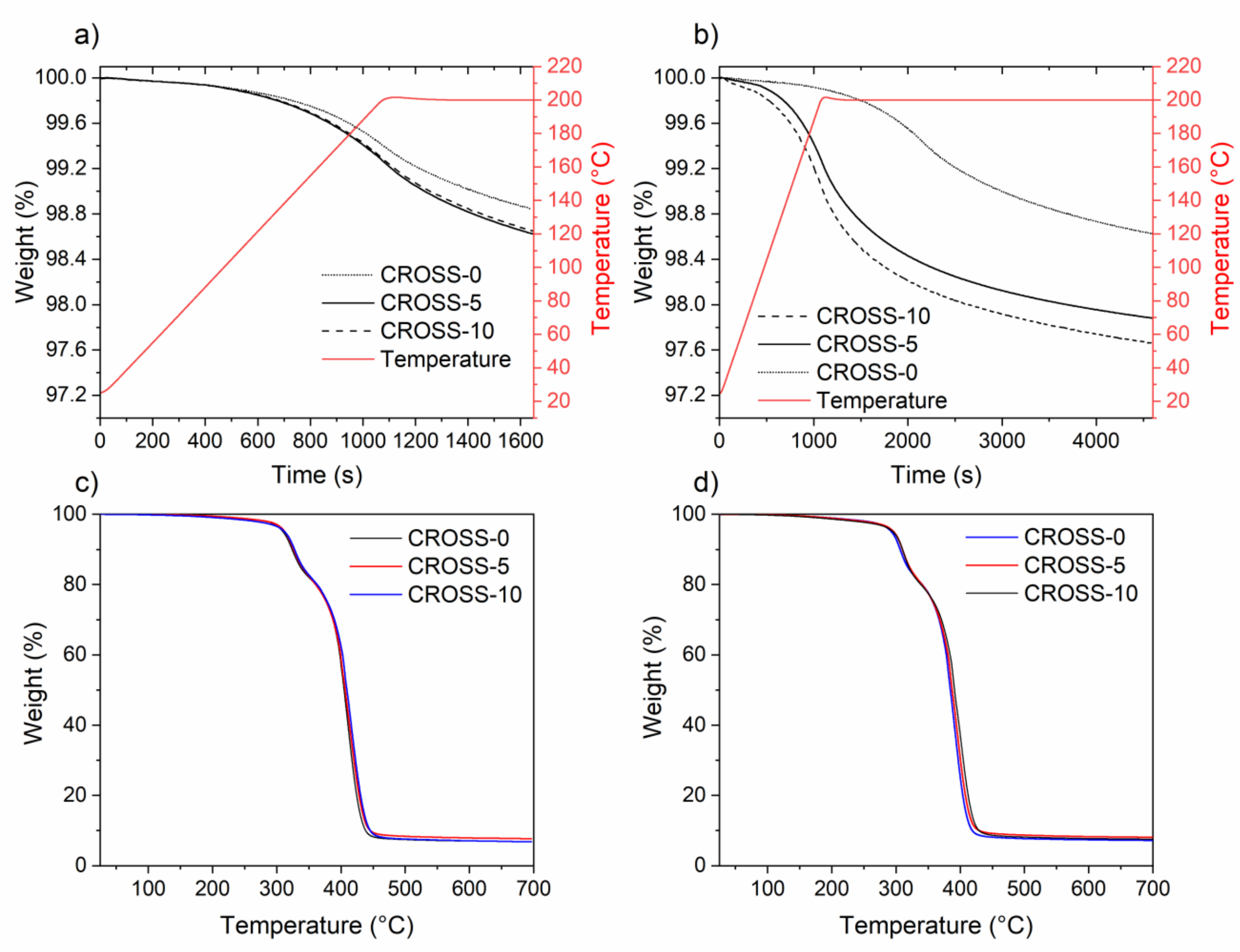

Figure S13. Thermogravimetric analysis of CROSS-0, CROSS-5, and CROSS-10. a) Isothermal TGA at 200 °C for 10 minutes. b) Isothermal TGA at 200 °C for 60 minutes. c) Temperature ramp from 25 to 700 °C at 10 °C/min. d) Temperature ramp from 25 to 700 °C at 3 °C/min.

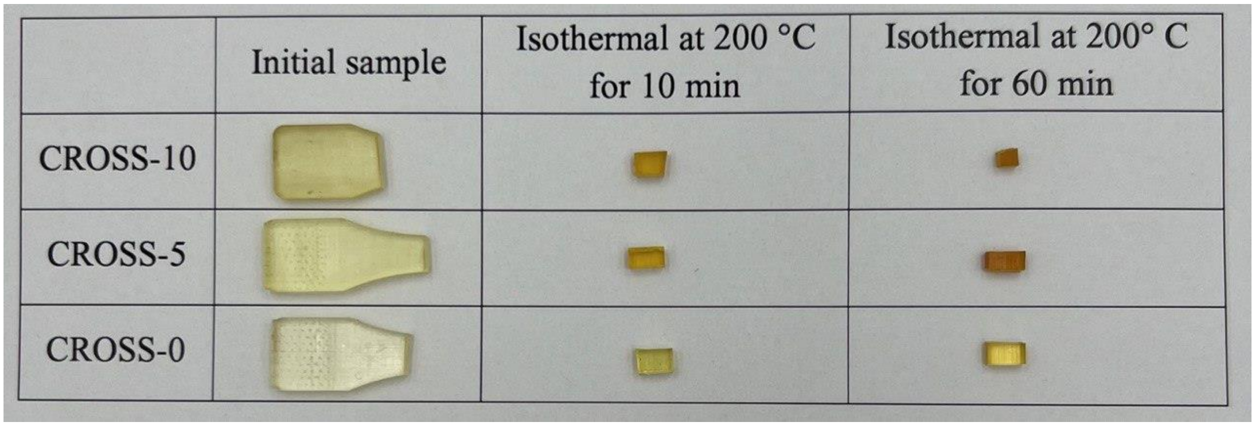

Figure S14. Photograph of the samples after TGA.

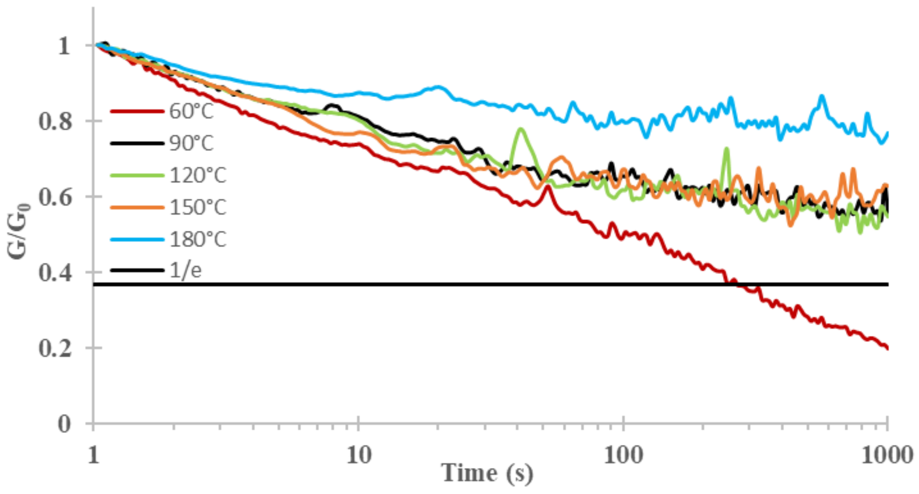

Figure S15. Normalized stress relaxation curves of printed resin Liqcreate Deep Blue.

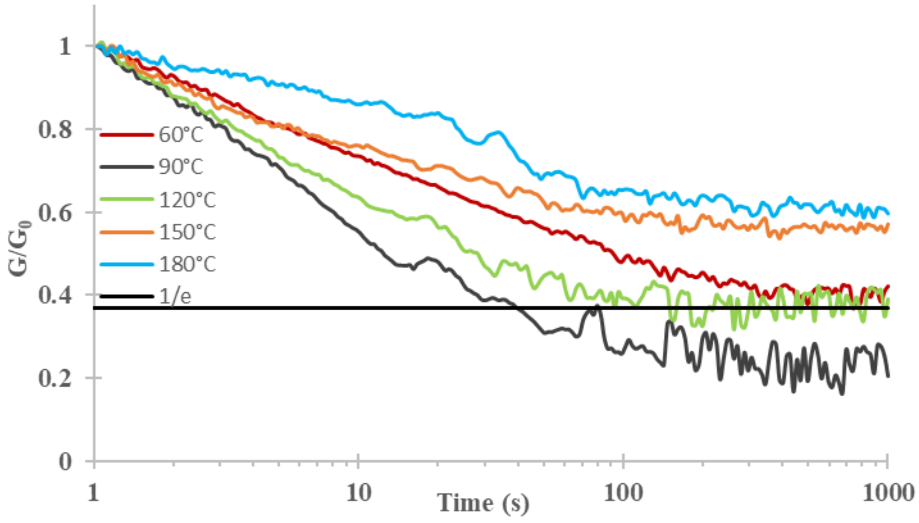

Figure S16. Normalized stress relaxation curves of the printed resin BMPR-06.

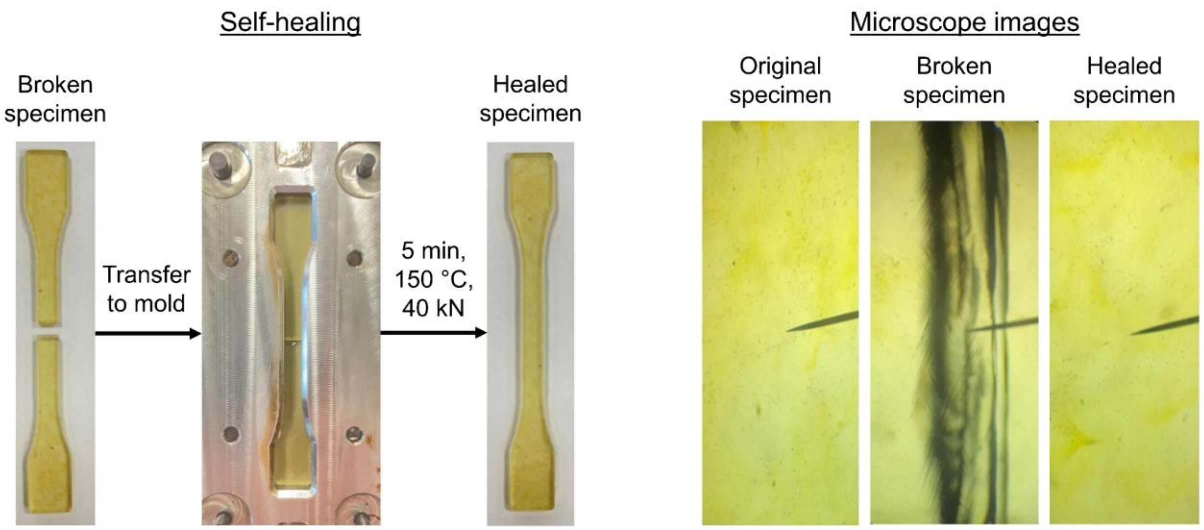

Figure S17. Self-healing experiment using a cross-linked tensile bar specimen from CROSS-0.

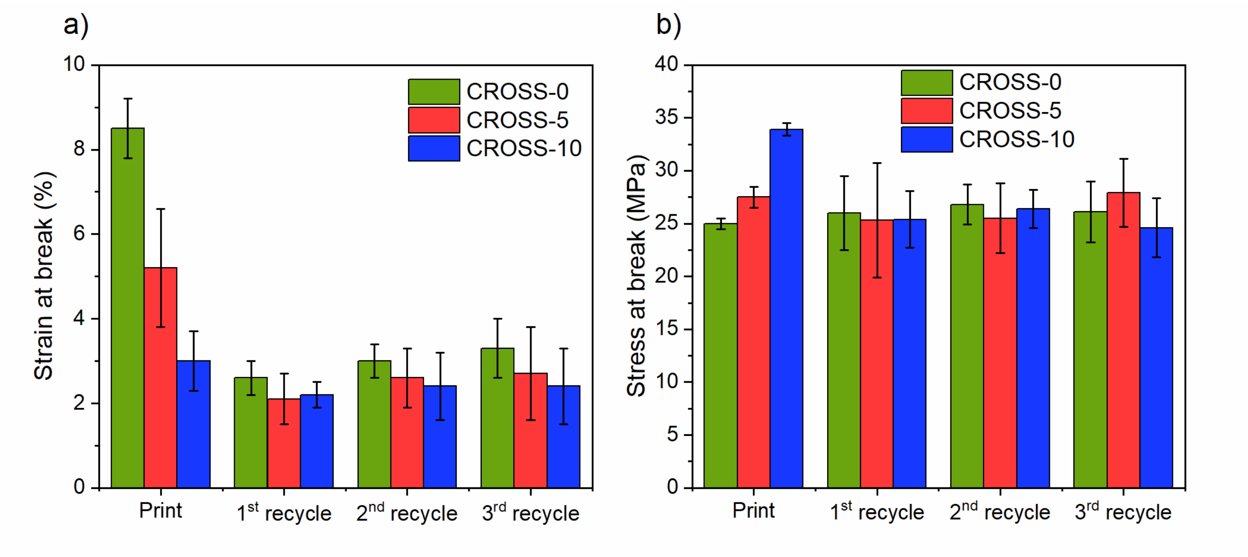

Figure S18. Bar graphs showing the tensile test results of CROSS-0, CROSS-5, and CROSS-10 with a) strain at break and b) stress at break.
